# The Tumorigenic Effect of the High Expression of Ladinin-1 in Lung Adenocarcinoma and Its Potential as a Therapeutic Target

**DOI:** 10.3390/molecules28031103

**Published:** 2023-01-22

**Authors:** Lei Hu, Yu Liu, Changfang Fu, Jiarong Zhao, Qianwen Cui, Qiuyan Sun, Hongqiang Wang, Li Lu, Haiming Dai, Xiaohui Xu, Wulin Yang

**Affiliations:** 1 Anhui Province Key Laboratory of Medical Physics and Technology, Institute of Health and Medical Technology, Hefei Institutes of Physical Science, Chinese Academy of Sciences, Hefei 230031, China; 2 Science Island Branch, Graduate School of University of Science and Technology of China, Hefei 230026, China; 3 School of Preclinical Medicine, Wannan Medical College, Wuhu 241002, China; 4 Biological Molecular Information System Laboratory, Institute of Intelligent Machines, Hefei Institutes of Physical Science, Chinese Academy of Sciences, Hefei 230031, China; 5 Department of Anatomy, Shanxi Medical University, Taiyuan 030024, China

**Keywords:** LUAD, LAD1, oncogenesis, prognosis, biomarker

## Abstract

The oncogenic role of Ladinin-1 (LAD1), an anchoring filament protein, is largely unknown. In this study, we conducted a series of studies on the oncogenic role of LAD1 in lung adenocarcinoma (LUAD). Firstly, we analyzed the aberrant expression of LAD1 in LUAD and its correlation with patient survival, tumor immune infiltration, and the activation of cancer signaling pathways. Furthermore, the relationship between LAD1 expression and K-Ras and EGF signaling activation, tumor cell proliferation, migration, and colony formation was studied by gene knockout/knockout methods. We found that LAD1 was frequently overexpressed in LUAD, and high LAD1 expression predicts a poor prognosis. LAD1 exhibits promoter hypomethylation in LUAD, which may contribute to its mRNA upregulation. Single-sample gene set enrichment analysis (ssGSEA) showed that acquired immunity was negatively correlated with LAD1 expression, which was verified by the downregulated GO terms of “Immunoglobulin receptor binding” and “Immunoglobulin complex circulating” in the LAD1 high-expression group through Gene Set Variation Analysis (GSVA). Notably, the Ras-dependent signature was the most activated signaling in the LAD1 high-expression group, and the phosphorylation of downstream effectors, such as ERK and c-jun, was strongly inhibited by LAD1 deficiency. Moreover, we demonstrated that LAD1 depletion significantly inhibited the proliferation, migration, and cell-cycle progression of LUAD cells and promoted sensitivity to Gefitinib, K-Ras inhibitor, and paclitaxel treatments. We also confirmed that LAD1 deficiency remarkably retarded tumor growth in the xenograft model. Conclusively, LAD1 is a critical prognostic biomarker for LUAD and has potential as an intervention target.

## 1. Introduction

Lung cancer is the most frequently diagnosed and fatal type of cancer worldwide, causing more than 1.8 million deaths each year [1,2]. Lung cancer is mainly classified into two types: small-cell lung cancer (SCLC), which accounts for 15% of the incidence, and non-small-cell lung cancer (NSCLC), accounting for the other 85% [3]. NSCLC can be divided into four main subtypes according to histopathology: lung adenocarcinoma (LUAD), lung squamous cell carcinoma (LUSC), large cell carcinoma, and undifferentiated NSCLC. Patients with NSCLC have a poor prognosis in the clinic, with only 15% surviving 5 years or more [4]. LUAD and LUSC are the most prevalent kinds, accounting for 90% of NSCLC cases [5], with LUAD accounting for approximately 40% of all cases [6]. The survival rate of patients with targeted EGFR or ALK gene mutations has improved in recent years [7]. However, the early stage of lung adenocarcinoma is relatively insidious, most patients are already in the middle and late stages at the time of diagnosis [8], and tumor cells can become resistant to targeted drugs in the clinic [9]. Thus, the lung adenocarcinoma mortality rate remains high [10]. As a consequence, it is very important to find novel diagnostic and prognostic markers and therapeutic targets to improve the survival of patients with LUAD.

The cytoskeleton is a functional component of cells and one of the most complex and versatile structures involved in phagocytosis, cell division, intracellular transport, cell adhesion, and signal transduction [11]. LAD1 (Ladinin-1) is a protein previously classified as collagen-anchored filaments in the basement membrane of mammalian epidermal cells [12,13]. It may contribute to the stability of the association of the epithelial layers with the underlying mesenchyme. At present, some articles have shown that LAD1 is expressed in some cancers, such as colorectal cancer [14], prostatic cancer [15], laryngeal cancer [16], and breast cancer [17], demonstrating that LAD1 expression is related to cancer migration and invasion. An analysis of METABRIC, a large clinical dataset of approximately 2000 breast cancer patients, demonstrates that a high abundance of the LAD1 transcript is associated with poor prognosis in breast cancer patients [18]. Further analysis showed that LAD1 expression was upregulated by multiple oncogenic signaling pathways [19,20]. A recent study demonstrated that upregulated LAD1 was involved in the progression of LUAD mediated by circ-ANXA7 [21]. Another study showed that LAD1 may be downstream of EGF signaling to regulate the oncogenic behaviors of mammary tumors [17]. Considering the importance of the EGF signaling pathway in the development of lung cancer, does LAD1 participate in EGF signaling to regulate lung cancer development? Although a comparative proteomic analysis found that LAD1 was abundant in lung adenocarcinoma [22], the exact role of LAD1 in LUAD remains to be explored.

The purpose of this study is to investigate the association of LAD1 with the prognosis of LUAD patients and the underlying carcinogenic mechanism. We investigated the LAD1 gene and protein expression in LUAD using transcriptomic and proteomic data from public databases, analyzed changes in gene promoter methylation and protein phosphorylation, and studied its relationship with tumor immune infiltrations and related carcinogenic signaling pathways. Further, we studied the effects of LAD1 on the proliferation, migration, cell cycle, and sensitivity to chemotherapeutic drugs in LUAD cells and tumorigenesis in nude mice.

## 2. Results

### 2.1. The Overexpression of LAD1 in LUAD

**Figure 1:**
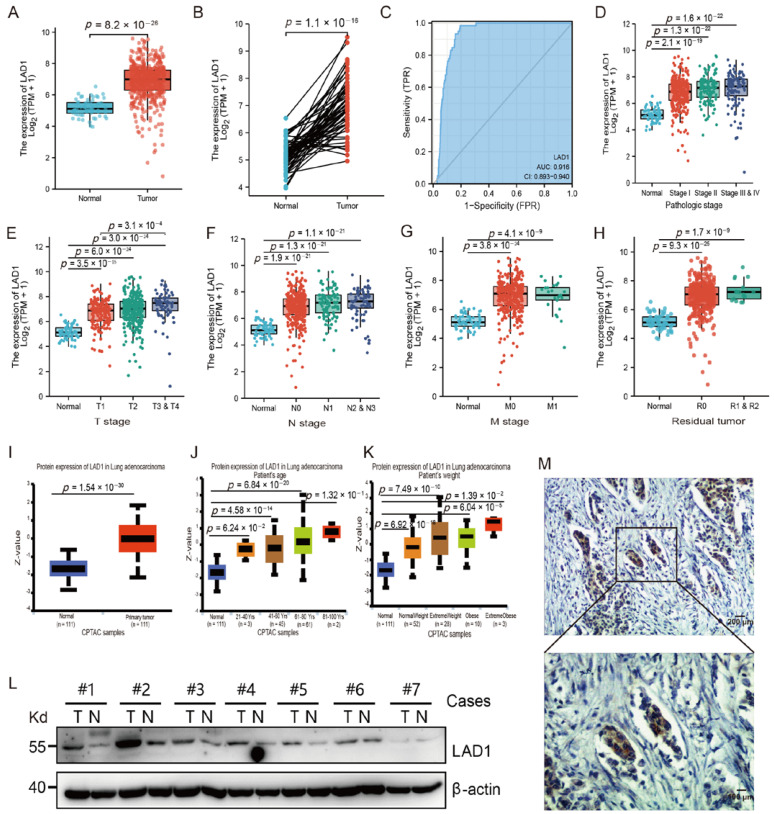
The upregulation of LAD1 expression in LUAD patients. (**A**) Comparison of LAD1 mRNA expression between 535 LUAD and 59 normal cases. (**B**) Comparison of LAD1 mRNA expression between 57 LUAD and matched normal tissues. (**C**) ROC analysis demonstrated the ability to differentiate tumors from normal tissues based on LAD1 expression level. (**D**–**H**) LAD1 expression in different groups of clinicopathological features, including pathologic stage (**D**), T/N/M stage (**E**–**G**), and residual tumor after chemotherapy (**H**). (**I**–**K**) LAD1 protein levels were elevated in LUAD patients and positively correlated with age and body weight. (**L**) The protein expression of LAD1 was examined by WB in 7 pairs of LUAD patients. #, Sample No. (**M**) Examination of LAD1 in LUAD tissues by immunohistochemistry.

The levels of LAD1 expression in 535 tumor tissues of LUAD were significantly higher than those in 59 normal tissues (*p* = 8.2 × 10^−26^, Figure 1A). It was also pronounced in 57 tumor tissues compared to matched normal tissues (*p* = 1.1 × 10^−16^, Figure 1B). Further, we investigated the ability to distinguish tumor tissues from normal ones based on LAD1 gene expression values. ROC analysis showed that the area under the curve (AUC) was 0.916, demonstrating good diagnostic sensitivity and specificity (Figure 1C). Additionally, we analyzed the correlation between LAD1 expression and various clinicopathologic features. The Wilcoxon rank-sum test showed that LAD1 expression increased significantly with the deterioration of clinical indicators such as clinical stages and T/N/M stages (Figure 1D–G). The high expression of LAD1 could also be detected in residual tumor tissues after treatment (Figure 1H). We evaluated the expression of the LAD1 protein using the CPTAC database. Consistent with the transcriptome data, the expression of LAD1 protein was elevated in the tumor tissue of LUAD (Figure 1I). Interestingly, the expression of LAD1 protein increased with age and body weight (Figure 1J,K). LUAD tumor tissues were also collected to confirm the elevated expression of LAD1 protein. Western blot results showed that LAD1 expression was upregulated in most tumor tissues (Figure 1L). The expression of LAD1 protein was further analyzed by immunohistochemistry and the results showed that LAD1 expression was strongly positive in the cytoplasm of lung adenocarcinoma cells (Figure 1M).

### 2.2. High Expression of LAD1 Predicts Poor Prognosis

**Figure 2:**
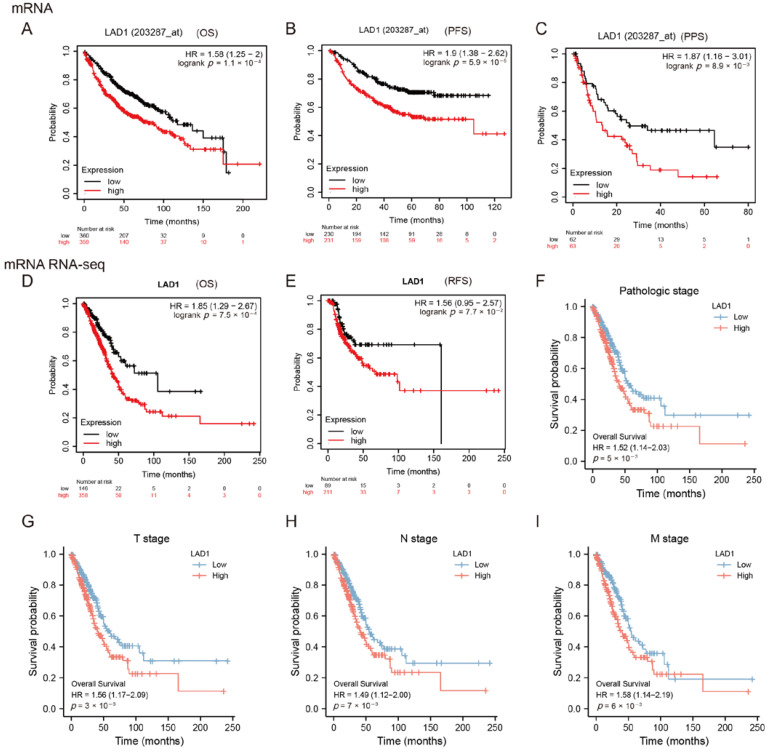
The upregulation of LAD1 in LUAD predicts poor prognosis. Kaplan–Meier analysis was performed based on gene chip data to compare the survival probability between high and low LAD1 expression groups, including overall survival (**A**), progression-free survival (**B**), and post-progression survival (**C**). (**D**) Kaplan–Meier curves based on mRNA RNA-seq data for overall survival analysis and relapse-free survival (**E**). (**F**–**I**) Subgroup analyses of overall survival based on high and low LAD1 expression, including pathologic stage (**F**), T stage (**G**), N stage (**H**), and M stage (**I**).

To evaluate the association between LAD1 expression and prognosis in LUAD, we first used gene-chip data by categorizing cancer cases into high- and low-expression groups. Kaplan–Meier survival analysis revealed that increased LAD1 expression was substantially more strongly associated with a poor prognosis than low LAD1 expression. As illustrated in Figure 2A–C, overall survival (OS) (*p* = 0.00011), progression-free survival (PFS) (*p* = 5.9 × 10^−5^), and post-progression survival (PPS) (*p* = 0.0089) were all significantly lower in the high-LAD1 group. Next, we used mRNA RNA-seq data (Figure 2D,E) to further elucidate that high expression of the LAD1 gene was associated with poor OS (*p* < 0.001) and relapse-free survival (RFS) (*p* = 0.077). Subgroup analysis showed that high LAD1 expression was associated with poor OS at different stages of disease classification, including the pathologic stage (*p* = 0.005, Figure 2F), the T stage (*p* = 0.0033, Figure 2G), the N stage (*p* = 0.007, Figure 2H), and the M stage (*p* = 0.006, Figure 2I). These results strongly suggest that LAD1 overexpression is a poor prognostic factor.

### 2.3. LAD1 Exhibits Promoter Hypomethylation and Protein Hyperphosphorylation in Lung Adenocarcinoma

**Figure 3:**
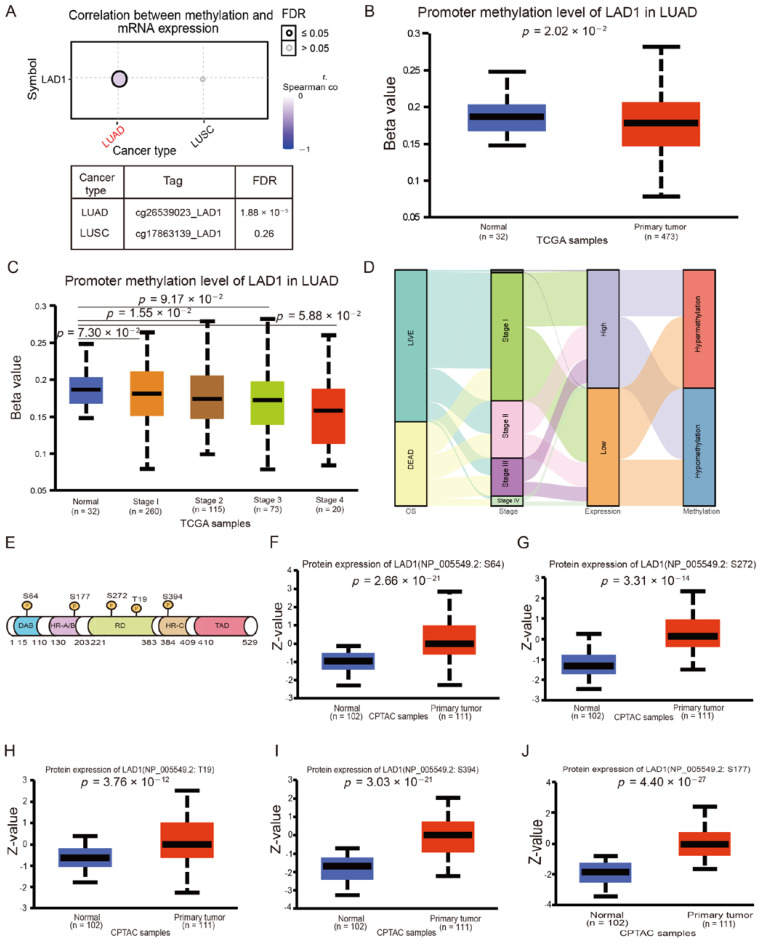
LAD1 exhibits promoter hypomethylation and protein hyperphosphorylation in LUAD. (**A**) The bubble plot shows the correlation between methylation and mRNA LAD1 expression in LUAD and LUSC. The size of the bubble point represents statistical significance. (**B**) The UALCAN database was used to analyze the methylation levels of the LAD1 gene promoter between LUAD tumor and normal samples, and the methylation levels were compared in different pathologic stages (**C**). (**D**) The Sankey diagram shows the relationship between survival status, tumor stage, mRNA expression, and promoter methylation. The path width of the lines represents the number of patients transferred from one state to another. (**E**) Potential phosphorylation modification sites of LAD1 were compared between normal and tumor samples in UALCAN, including S64 (**F**), S272 (**G**), T19 (**H**), S394 (**I**), and S177(**J**).

We analyzed the link between promoter methylation and gene expression using the GSCALite database. As shown in Figure 3A, there was a significant negative correlation between LAD1 expression and methylation levels in LUAD, but not in LUSC, another lung cancer subtype (Figure 3A, *p* = 1.88 × 10^−5^). Similar results were obtained using the UALCAN database, confirming the hypomethylation of the LAD1 promoter in LUAD (Figure 3B,C). These results suggest that the high expression of LAD1 in LUAD tumor tissue may be related to promoter hypomethylation. To examine the clinical outcomes linked with LAD1 methylation and expression, we analyzed data from 535 individuals, including information about methylation, expression, and survival characteristics in the TCGA database. A Sankey diagram was used to display the detailed routes and patterns across tumor stages, promoter methylation levels, LAD1 mRNA expression, and survival status. It was established that the hypomethylation of the LAD1 promoter is tightly linked with LAD1 mRNA overexpression, ultimately resulting in poor prognoses in patients with LUAD (Figure 3D). Posttranslational phosphorylation modification is always a common molecular mechanism for LAD1-mediated EGF–ERK signaling [17]. Therefore, we used the CPTAC database to analyze differences in LAD1 phosphorylation levels between primary tumor tissues and normal tissues. LAD1 was found to be highly phosphorylated at five sites in LUAD, including S64, S272, S394, S177, and T19 (Figure 3E–J). Thus, the hyperphosphorylation of LAD1 may be related to the occurrence and development of lung adenocarcinoma. Collectively, epigenetic and posttranscriptional modifications of LAD1 in lung adenocarcinoma may be beneficial to tumorigenesis.

### 2.4. The Correlation between LAD1 Expression and Immune Infiltration

**Figure 4:**
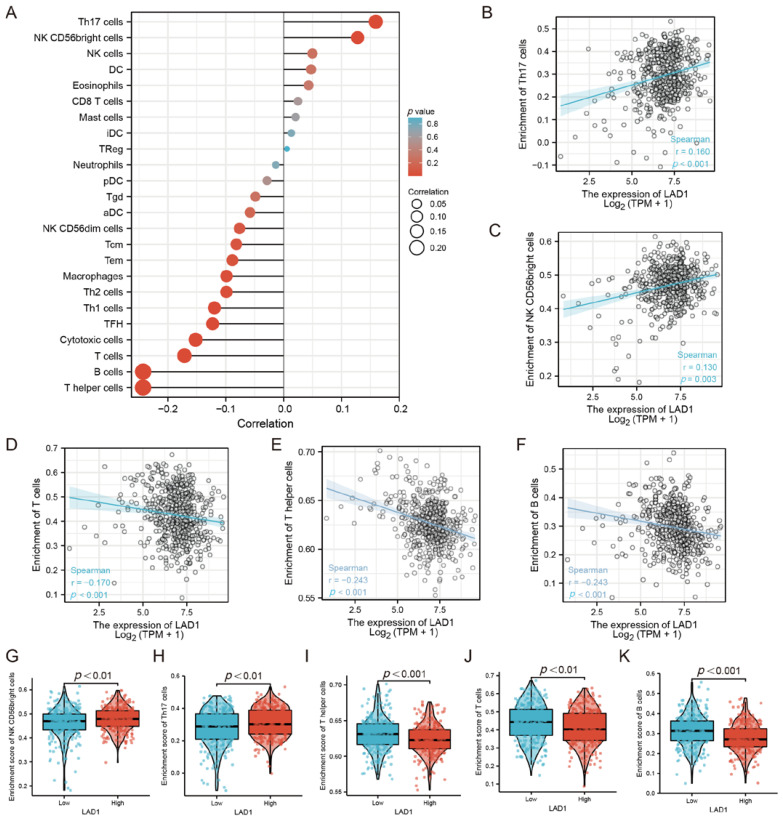
The relationship between LAD1 expression and immune infiltration. (**A**) The correlation between the relative abundance of 24 immune cells and LAD1 expression. The dot sizes represent the absolute Spearman’s correlation coefficient values. LAD1 expression was positively correlated with the abundance of innate immune cells, such as Th17 cells (**B**) and NK CD56 bright cells (**C**), and negatively correlated with acquired immune cells, such as T cells (**D**), T helper cells (**E**), and B cells (**F**). NK CD56 bright cells and Th17 cells were enriched in the LAD1 high-expression group (**G**,**H**); T helper cells, T cells, and B cells were enriched in the LAD1 low-expression group (**I**–**K**).

LAD1, an anchoring filament protein, is generally considered to be a component of the basement membrane zone and is also recognized as a filamin-binding regulator of actin dynamics in response to EGF signaling [17,23]. We speculated that LAD1 was involved in the regulation of the tumor microenvironment and was related to immune cell infiltration. The ssGSEA method was used to evaluate the infiltration of 24 immune cell types in LUAD (Figure 4A), and it could be seen that the infiltration degree of most immune cell types was negatively correlated with LAD1 expression. Specifically, LAD1 expression was mainly positively correlated with innate immune cell infiltration, such as Th17 cells (Figure 4B), NK CD56 bright cells (Figure 4C), etc., while being negatively correlated with acquired immune cell infiltration, such as T cells (Figure 4D), T helper cells (Figure 4E), B cells (Figure 4F), etc. Accordingly, innate immune cells (NK CD56 bright cells, Th17 cells) were enriched in the LAD1 high-expression group (Figure 4G,H), while acquired immune cells (T helper cells, T cells, B cells) were enriched in the LAD1 low-expression group (Figure 4I–K).

### 2.5. GSVA Identified LAD1 Overexpression Relates with “K-Ras Addiction” Phenotype

**Figure 5:**
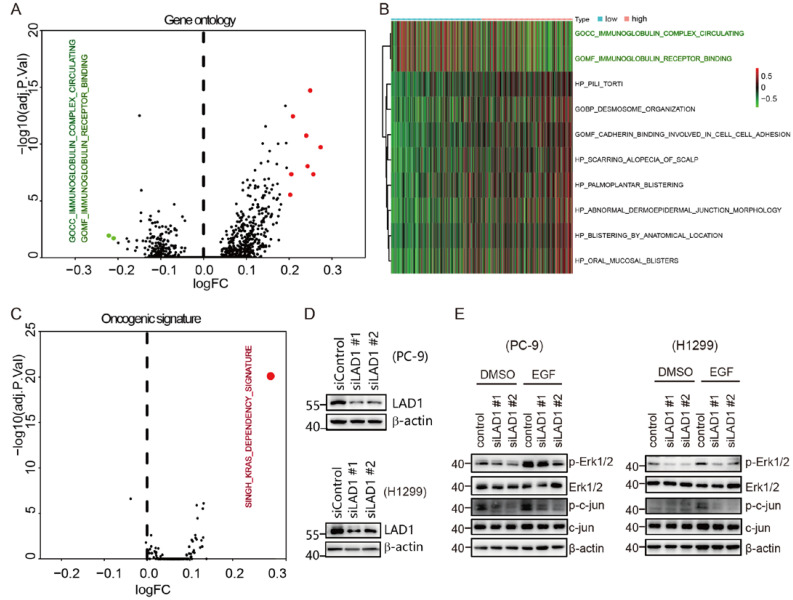
GSVA analysis revealed differences in gene ontology categories and oncogenic signatures between LAD1 high-expression and low-expression groups. (**A**) The differences in gene ontology categories between the LAD1 high- and low-expression groups shown by a volcanic map. (**B**) The heatmap shows the gene ontology categories that differ significantly. (**C**) The differences in oncogenic signatures between the LAD1 high- and low-expression groups shown by a volcanic map. The SINGH_KRAS_DEPENDENCY_SIGNATURE was the only oncogenic signature with a significant difference. (**D**) LAD1 knockdown by two small interfering RNAs (siLAD1 #1 and siLAD1 #2) in PC-9 and H1299 was examined by Western blot. (**E**) The levels of ERK1/2, c-jun, phosphorylated ERK1/2, and c-jun were examined in control and LAD1-knockout PC-9 cells, with or without EGF treatment.

Since LAD1 overexpression leads to poor prognosis and may be related to the activation of oncogenic signals, we used GSVA to study the differences in gene ontology, KEGG pathways, and oncogenic signatures between the high- and low-expression groups of LAD1. No KEGG pathway enriched in LAD1 high-expression group was found, but there were differences in gene ontologies between the LAD1 high- and low- expression groups (Figure 5A). Particularly, we found that terms of GOCC_IMMUNOGLOBULIN_COMPLEX_CIRCULATING and GOMF_IMMUNOGLOBULIN_RECEPTOR_BINDING were significantly downregulated in the LAD1 high-expression group (Figure 5B), suggesting immune tolerance in correlation with LAD1 upregulation. This may be the reason why LAD1 expression is negatively correlated with immune infiltration. The upregulation of SINGH_KRAS_DEPENDENCY_SIGNATURE was the only activated oncogenic signature in the LAD1 high-expression group (Figure 5C), suggesting that the overexpression of LAD1 may facilitate the activation of K-Ras, resulting in the so-called “K-Ras addiction” of tumor cells [24]. Previous studies have suggested that LAD1 is a filamin-binding regulator of actin dynamics responding to EGF and is involved in the EGF-to-ERK cascade [17]. EGF and K-Ras signals always interact to regulate downstream ERK activation [25]. We hypothesized that LAD1 overexpression-mediated K-Ras addiction in lung adenocarcinoma may integrate with EGF signaling and influence its common downstream effector. We used PC-9 and H1299 cells to examine the effect of LAD1-knockdown on ERK and c-jun, two common downstream effectors for K-Ras and EGF signaling (Figure 5D). As shown in Figure 5E, ERK and c-jun protein levels were not heavily affected upon LAD1 knockdown, but their phosphorylation levels were markedly reduced in LAD1-deficient cells, particularly upon EGF treatment, suggesting that LAD1 may mediate the interplay and transmission of EGF and K-Ras signals in lung adenocarcinoma cells.

### 2.6. Critical Role of LAD1 in LUAD Cell Migration, Proliferation, Cell Cycle, and Apoptosis

**Figure 6:**
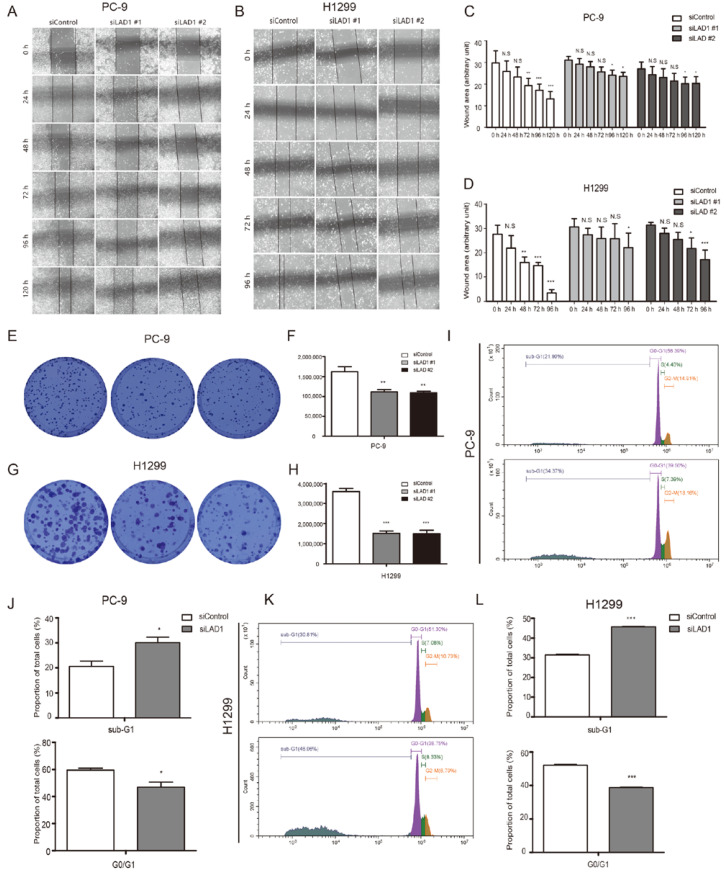
The critical role of LAD1 in LUAD cell migration, proliferation, cell cycle, and apoptosis. (**A**–**D**). A wound-healing assay was performed in control (siControl) and LAD1-knockdown (siLAD1) PC-9 (**A**,**C**) and H1299 (**B**,**D**) cells. Representative images were obtained at the indicated time points, and the results were quantified with Image J software. Error bars, means ± SD (* *p* < 0.05, ** *p* < 0.01, *** *p* < 0.001 versus control, n = 3 independent experiments). (**E**–**H**) Control and LAD1-knockdown PC-9 and H1299 were subjected to a colony-formation assay. Representative images for colony growth are shown (**E**,**G**), along with the quantification of the colonies using Image J software (**F**,**H**). Error bars, means ± SD (** *p* < 0.01 and *** *p* <0.001, n = 3 independent experiments). (**I**–**L**). Control and LAD1-knockdown PC-9 and H1299 were subjected to PI staining and flow cytometry detection. Representative images were shown (**I**,**K**), and results were quantified with GraphPad Prism 5 (**J**,**L**). Error bars, means ± SD (* *p* < 0.05, ** *p* < 0.01, *** *p* < 0.001 vs. control, n = 3 independent experiments).

The knockdown of LAD1 in PC9 and H1299 was applied to study cell migration and invasion. A wound healing assay showed that LAD1 knockdown significantly inhibited the migratory ability of PC-9 and H1299 cells. Control cells closed the wound rapidly after culturing for some time, while the LAD1-knockdown cells still left an obvious unhealed wound area (Figure 6A–D). In addition, colony formation assays were also performed to detect the proliferation of LAD1-knockdown cells. As shown in Figure 6E–H, LAD1 knockdown significantly reduced the number of formed cell colonies, with a relatively smaller size in comparison with control cells. Furthermore, we stained PC-9 and H1299 cells with propidium iodide (PI) and analyzed the cell cycle by flow cytometry. The results showed that after LAD1 was knocked down, the cell cycle was arrested in the G0/G1 phase, and especially the number of cells in the sub-G1 phase increased significantly (Figure 6I–L), suggesting that LAD1 may be involved in the regulation of apoptosis. In summary, LAD1 might be critical for the migration, proliferation, cell cycle, and apoptosis of LUAD cells.

### 2.7. LAD1 Upregulation Contributes to Chemotherapeutic Drug Resistance in LUAD Cells

**Figure 7:**
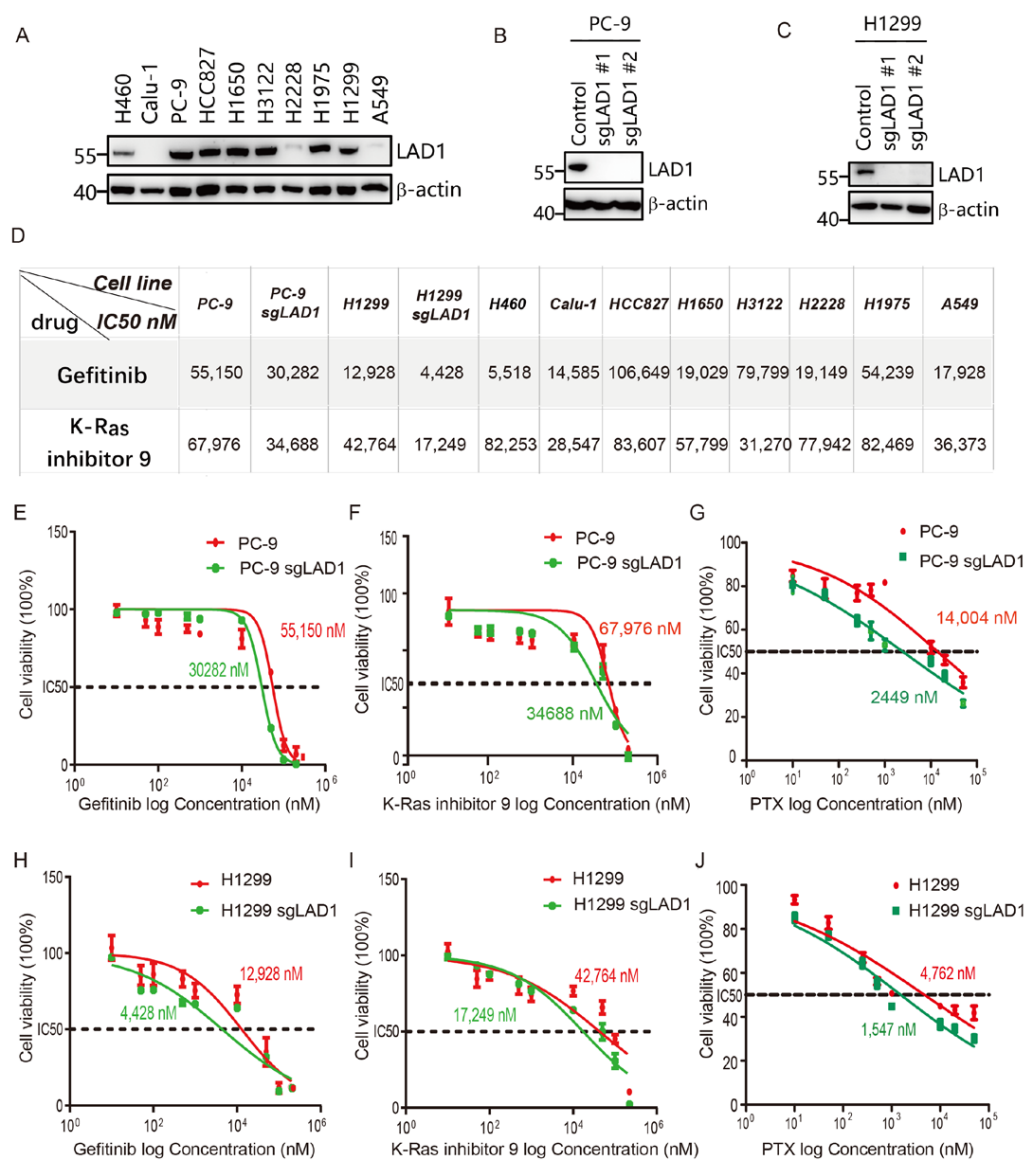
LAD1 contributes to drug resistance in LUAD cells. (**A**) Expression levels of LAD1 as detected by Western blot in LUSC, LCLC, and LUAD cell lines. β-actin was used as a loading control. (**B**,**C**) LAD1 knockout by two sgRNAs (sgLAD1 #1 and sgLAD1 #2) in PC-9 and H1299 was examined by Western blot. (**D**) A summary of the CCK8 assay was used to test the cell viability upon drug treatment. Each experiment was repeated three times. The IC50 values of control and LAD1-knockout PC-9 (**E**–**G**) and H1299 (**H**–**J**) cells were determined by CCK8 assay. Results were analyzed with GraphPad Prism 5. Error bars, means ± SD (*n* = 3 independent experiments).

The above results showed that the high expression of LAD1 predicted poor outcomes in LUAD and affected K-Ras and EGF signaling, suggesting that the high expression of LAD1 may affect the chemotherapeutic drug sensitivity of lung cancer cells. We firstly tested LAD1 expression in ten lung cancer cell lines, including one LUSC, one LCLC (large cell lung cancer), and eight LUADs. LAD1 was highly expressed in six of the eight LUAD cell lines and under-expressed in two of them, while there was low or no expression in the LCLC and LUSC cell lines (Figure 7A). Then, we treated these ten cell lines with Gefitinib, an EGFR tyrosine kinase inhibitor, or K-Ras inhibitor 9, which blocks the formation of GTP-K-Ras to inhibit its activation. As summarized in Figure 7D, cells with low LAD1 expression, including H460, Calu-1, H2228, and A549, showed more sensitivity to Gefitinib than high-expressing cells except for H1299 (Supplementary Figure S1). Similarly, two LAD1 low-expressing LUAD cells, Calu-1 and A549, showed more sensitivity to K-Ras inhibitor 9 (Supplementary Figure S2). To further evaluate the role of LAD1 in drug resistance, we constructed LAD1-specific knockout cell lines PC-9 (Figure 7B) and H1299 (Figure 7C) by an sgRNA (single-guide RNA)-guided CRISPR system. As expected, LAD1 depletion significantly reduced the IC50 of Gefitinib, K-Ras inhibitor 9, in both PC-9 (Figure 7E,F) and H1299 cells (Figure 7H,I), as summarized in Figure 7D. We also compared the sensitivity of wild-type and LAD1-knockout PC-9 and H1299 cells to a widely used chemotherapy agent, paclitaxel (PTX), which induces cytotoxicity by interfering with the physiological function of microtubules and the process of cell mitosis. As shown in Figure 7G–J, LAD1 depletion reduced the IC50 of PTX from 14,004 nM to 2,449 nM and 4,762 nM to 1,547 nM in PC-9 and H1299, respectively. Taken together, these results suggest that LAD1 is an important factor in the development of drug resistance in LUAD cells, and therefore may be a potential intervention target to improve drug sensitivity.

### 2.8. LAD1 Depletion Reduces Tumorigenicity of LUAD Cells in the Xenograft Model

**Figure 8:**
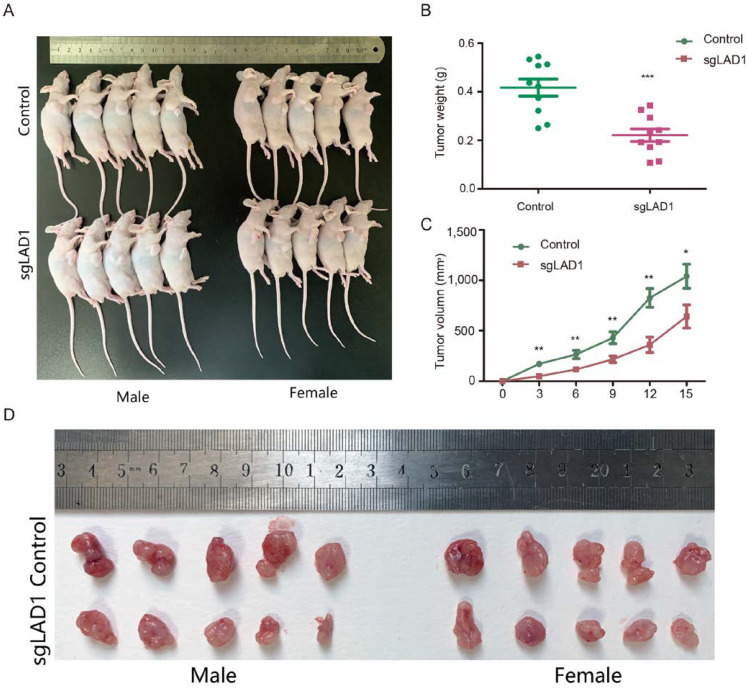
LAD1 depletion reduces tumorigenicity in the xenograft model. Control and mixed LAD1-knockout cells (sgLAD1 #1 and #2 mixed at 1:1) were separately inoculated subcutaneously into Balb/c nude mice (**A**). The tumor sizes were quantified at different time points after inoculation (**B**). Two weeks after implantation, the tumors were weighed (**C**), and images of the transplanted tumors are displayed (**D**). Error bars, means ± SD (* *p* < 0.05, ** *p* < 0.01, *** *p* < 0.001 vs. control).

To verify whether LAD1 was involved in modulating LUAD tumor growth in vivo, we implanted wild-type and LAD1-knockout PC-9 cells subcutaneously in Balb/c nude mice. Two weeks later, the mice were sacrificed, and tumor tissues were collected (Figure 8A). The results showed that the volume and weight of the tumor masses in nude mice injected with control cells were almost two times larger than in those with the LAD1-depleted xenografts (Figure 8B–D), indicating that LAD1 is required for LUAD cell growth in vivo.

## 3. Discussion

In this paper, we found that LAD1 expression was elevated in LUAD, associated with unfavorable prognosis, and negatively correlated with acquired immunity. GSVA analysis showed that the overexpression of LAD1 led to the upregulation of a Ras-dependent onco-signature, which may make tumor cells “K-Ras addicted”. It was also demonstrated that the phosphorylation of ERK and c-jun, the common downstream molecules of Ras and EGFR signals, was indeed inhibited by LAD1 knockdown. Furthermore, we demonstrated that LAD1 knockdown significantly inhibited the proliferation, migration, and cell cycle progression of LUAD cells and promoted sensitivity to paclitaxel treatment. The xenograft model experiment proved that LAD1 knockout remarkably retarded tumor growth in vivo.

LAD1, which was cloned from a mouse skin cDNA library, seems to be a secreted protein that functions as a component of the basement membrane [23]. It is located on chromosome 1, and its exon region is well-conserved in mammals. The encoded protein harbors no recognizable structural domains, other than an arginine-rich N-terminal stretch and six serine–glutamate–lysine (SEK) tripeptide motifs, the function of which remains largely unknown [26]. So far, there are few functional studies on LAD1. A recent study found that LAD1 is associated with malignant and aggressive human breast tumors, and the authors have proved through in vitro studies, animal models, and clinical data that the widespread expression of LAD1 acts as a substrate for the downstream phosphorylation of the EGF pathway [17,27]. Our work shows that LAD1’s high expression is associated with a “K-Ras addiction” phenotype. The knockdown experiment proved that LAD1 does affect the phosphorylation of the ERK protein downstream of EGFR, indicating that LAD1 does play a role in EGF-stimulated signal transduction. A previous study demonstrated that EGF could promote breast cancer cell survival by regulating the expression of anti-apoptotic factor Mcl1 through the MAPK-Elk1 signaling pathway [28]. Thus, theoretically, LAD1 could also be associated with anti-apoptotic signaling via EGF-Elk1 signaling. However, the exact mechanism still needs further research.

In recent years, with the development of molecular biology and in-depth research on the pathogenesis of cancer, antitumor drugs targeting specific molecules have emerged. Epidermal growth factor receptor (EGFR) is overexpressed in a variety of tumors [29]. It plays an important role in regulating the growth, repair, angiogenesis, invasion, and metastasis of tumor cells, and has become one of the important targets of anti-tumor drugs [30,31]. EGFR gene mutation is a form of the gene variant that frequently occurs in lung cancer, resulting in an overactivated EGF signal that stimulates abnormal cell growth and tumorigenesis [32,33]. EGFR mutations are present in approximately 15% of patients with non-small-cell lung cancer. Drugs that target specific EGFR mutations have significantly extended the survival of lung cancer patients [34]. However, after the use of targeted drugs for a period of time, EGFR may generate new mutations or the activation of other oncogenic signaling pathways, resulting in drug resistance [35]. Therefore, fundamentally blocking oncogenic signaling pathways may be a real effective measure for the treatment of lung cancer. In this work, we found that the overexpression of LAD1 causes “K-Ras addiction”, including the activation of the EGF–ERK signal, which can be blocked by LAD1 knockdown. Moreover, we found that LAD1 knockout significantly promotes the sensitivity of PC-9 and H1299 to Gefitinib, K-Ras inhibitor 9, and PTX treatment, suggesting that LAD1 is involved in chemotherapeutic drug resistance in LUAD. Therefore, the intervention in LAD1 may be a potential treatment direction for lung cancer.

## 4. Materials and Methods

### 4.1. Data Source and Processing

TCGA data were downloaded from the UCSC Xena database (http://xena.ucsc.edu/ (accessed on 7th August 2021)) [36], including information for RNA-seq (RSEM TPM) and clinical profiles (including both phenotype and survival data). Expression levels were normalized using the z-score of log_2_ (TPM + 0.001) to exclude potential bias. Unavailable or unknown clinical features were regarded as missing values.

### 4.2. Differential Expression Analysis of LAD1

Boxplots and scatter plots were generated to calculate the differential expression of LAD1 between tumor and normal cases. The diagnostic performance of LAD1 was calculated using receiver operating characteristic (ROC) curves by the pROC and the ggplot2 packages in R software. LAD1 expression above or below the median expression value was defined as LAD1-high or LAD1-low, respectively.

### 4.3. Survival Analysis

Kaplan–Meier graphs were constructed using the KM-plotter database (http://kmplot.com/ (accessed on 7th August 2021)) [37], which is handled by a PostgreSQL server that integrates gene expression and clinical data simultaneously. This database contains gene expression data and relapse-free and overall survival information from GEO (Affymetrix microarrays only), EGA, and TCGA. To analyze the prognostic value of LAD1, patient samples were split into two groups according to mean expression value, then compared by a Kaplan–Meier survival plot, and the hazard ratio with 95% confidence intervals and log-rank p-values were also calculated.

### 4.4. Genome-Wide Analysis of LAD1

The UALCAN portal (http://ualcan.path.uab.edu/analysis-prot.html (accessed on 9 August 2021)), an interactive web resource for analyzing cancer omics data, integrates the CPTAC (clinical proteomic tumor analysis consortium) dataset for protein expression analysis [38]. We explored the expression levels of the LAD1 protein, phosphoprotein (with phosphorylation at the S64, S177, S272, T19, and S394 sites), and the methylation level of LAD1 mRNA between tumor and normal tissues, respectively. The correlation between methylation and mRNA expression was performed by an interactive web tool, GSCALite [39].

### 4.5. Gene Set Variation Analysis (GSVA)

The GSVA was applied to explore the differences in oncogenic signature, KEGG, and gene ontology between the high and low LAD1 expression groups using the corresponding gene sets (https://www.gsea-msigdb.org/gsea/msigdb (accessed on 10th August 2021)) and analyzed by the R package “GSVA” combined with “limma”. A functional term with adjusted *p*-value < 0.05 and log_2_ (fold change) > 0.2 was considered statistically significant for enrichment.

### 4.6. Analysis of Immune Cell Infiltration

The relative tumor infiltration levels of 24 immune cell types were quantified by ssGSEA to interrogate the expression level of signature genes in published gene lists [40]. To explore the correlation between LAD1 and the infiltration levels of immune cells and the associations between the infiltrations of immune cells and the different expression groups of LAD1, the Wilcoxon rank sum test and Spearman correlation were adopted.

### 4.7. Cell Lines

The human cell lines used in this study included lung squamous cell carcinoma cell line Calu-1, large-cell lung cancer cell line H460, lung adenocarcinoma cell lines A549, PC-9, H1229, HCC827, H3122, H1650, H2228, and H1975, and embryonic kidney cell line 293T. Calu-1, H1299, H460, and H1975 were kind gifts from Han Wei (Hefei Institutes of Physical Science, Chinese Academy of Sciences (CASHIPS)). HCC827 and H1650 were kindly gifted by Liu Qingsong (CASHIPS). A549 and PC-9 were obtained from Chu Yannan (CASHIPS). H3122 and H2228 were acquired from Wang Meng, and 293T was purchased from CCTCC (China Center for Type Culture Collection). Among them, Calu-1 and 293T were cultured in DMEM, A549 was cultured in Ham’s F-12K, and all other cells were cultured in RPMI-1640. All the cell culture mediums were supplemented with 10% FBS (fetal bovine serum, FB25015, Clark) and 1% PS (penicillin–streptomycin, SV30010, Hyclone) and maintained at 37 °C with 5% CO_2_.

### 4.8. Specimen Collection and Immunohistochemistry

Resected lung adenocarcinoma tissue and the adjacent tissue specimens were obtained from the Hefei Cancer Hospital, Chinese Academy of Sciences (CAS). The study protocols were approved by the Institutional Review Board of the Hefei Institutes of Physical Science, CAS (Y-2019-21). For immunohistochemistry, tissues were fixed in formalin for at least 12h, followed by dehydration with different grades of alcohol and chloroform–alcohol mixture, and embedded in paraffin. The resultant formalin-fixed paraffin-embedded (FFPE) blocks were cut into 2 μm tissue sections. To conduct IHC, tissue sections were de-paraffinized with xylene, rehydrated with various grades of alcohol (100%, 95%, 80%, and 70%), blocked with serum, then incubated with H_2_O_2_ reagent to quench endogenous peroxidase. Subsequently, the sections were successively incubated with LAD1 antibody, secondary antibody, and DAB substrate for color rendering, and photographed under a microscope.

### 4.9. Western Blotting

Tissue samples were firstly cut into tiny pieces using a razor blade and transferred to a mortar to grind to a fine powder. Both the powder and cells were lysed by RIPA lysis buffer (P0013c, Beyotime) with a protease inhibitor cocktail (04693132001, Roche) and then centrifuged (12,000 g) at 4 °C for 15 min to harvest the supernatants. Equal amounts of cell lysate (50 μg) measured by BCA protein assay kit (P0011, Beyotime) were then separated by SDS-PAGE and transferred to PVDF membranes followed by blocking with 5% skim milk. Then, the membrane was incubated overnight with the indicated primary antibody at 4 °C, followed by incubation with HRP-conjugated secondary antibodies for 2 h at room temperature. All proteins were visualized with the Tanon High-sig ECL Western Blotting substrate (180-501, Tanon, Shanghai, China).

### 4.10. LAD1 Knockdown and Knockout

Two individual siRNAs targeting the LAD1 gene and nontargeting control siRNA were purchased from GenePharma (Shanghai, China). The sequences of siLAD1 #1 and siLAD1 #2 were 5′-CAGUGAAGUUGGGAGAGAA-3′ and 5′-CAGACAACACAGUGAAGUU-3′, respectively. The sequence of siControl was 5′-UUCUCCGAACGUGUCACGU-3′. For siRNA knockdown, 1.5/6 × 10^5^ PC-9 or H1299 cells were seeded in the 24/6-well plates. After 24 h, 20/80 pmol of siRNAs were transfected using Lipofectamine RNAiMAX (13778-075, Invitrogen) according to the manufacturer’s protocol. Briefly, 20/80 pmol siRNAs and 2/8 μL RNAiMAX were separately diluted in 50/200 μL Opti-MEM (31985-070, Gibco) medium without serum. After 10 min, the RNAiMAX was transferred to the siRNAs and mixed gently. After 15 min incubation at room temperature, the mixtures were added and incubated with cells for 8h followed by the substitution of the medium with fresh RPMI-1640 mediums supplemented with 10% FBS and 1% PS and maintained at 37 °C with 5% CO_2_ for further treatment.

For LAD1 knockout, two gRNAs (guide RNAs), sgLAD1 #1 (5′-GAGCTCCACCACGGACGATG-3′) and sgLAD1 #2 (5′-GGCTCAGCCAGAATGGAGAC-3′), were cloned into lentiCRISPR v2. Then, lentiCRISPR v2-sgLAD1 #1/#2, together with the packing plasmids pMD2G and pSPAX2, were transfected into 293T cells in a ratio of 5:2:3. After 48 h and 72 h, cell supernatants containing the lentivirus were collected and used to infect PC-9 and H1299 cells for another 24 h. The infected positive cells were then sorted by puromycin (HY-B1743A, MedChemExpress) and verified by Western blotting.

### 4.11. Cell Cycle Analysis

The cell cycle distribution was determined by CytoFLEX (Beckman Coulter). Briefly, 2 × 10^5^ WT or LAD1-knockout (sgLAD1 #1 and #2 mixed at 1:1) PC-9 or H1299 cells were seeded into 24-well plates. After 24 h, cells were collected and fixed with cold 70% ethanol (500 μL) and stored for 2h at 4 °C, then centrifuged at 1000 rpm for 10 min to collect the cells. After washing three times with cold PBS, 10 μg/mL propidium iodide (C0080, Solarbio) was added to the cells and incubated for 15 min at room temperature. DNA content was detected by CytoFLEX (Beckman Coulter, Inc., Brea, CA, USA) and analyzed by CytExpert software (Beckman Coulter, Inc., Brea, CA, USA).

### 4.12. Reagents and Cell Viability Assay

Gefitinib (T1181, Topscience, Shanghai, China), K-Ras inhibitor 9 (T8756, TargetMol, Boston, MA, USA), and paclitaxel (PTX, T0968, TargetMol, Boston, MA, USA) were dissolved in dimethyl sulfoxide (DMSO, Sigma-Aldrich, Schnellendorf, Germany) at a stock concentration of 20 mM. For cell viability assay, 5000 cells were seeded into a 96-well plate. After 24 h, DMSO or various concentrations of drugs were added to the medium and treated for another 24 h. Cell viability was then detected by Cell Counting Kit-8 (C0039, Beyotime) according to the manufacturer’s instructions. Briefly, 10 μL CCK-8 solution was added and incubated for 2h at 37 °C, and absorbance at 450 nm was measured using CMax Plus (Molecular Devices, LLC). The half-maximal inhibitory concentration (IC50) values were generated and compared using GraphPad Prism. Each sample was tested in triplicate to obtain the average. Data are shown as means ± (standard deviations) SDs.

### 4.13. CCK8 Assay

Cell sensitivity to paclitaxel (PTX) was determined using the CCK-8 assay (C0039, Beyotime). Briefly, 5000 cells were seeded into a 96-well plate for about 24 h until the confluence reached 30–40%. Various concentrations of PTX (from 10 nM to 10 μM) were added and incubated at 37 °C with 5% CO_2_ for a further 12, 24, 36, and 48 h. Then 10 μL CCK-8 solution was added and incubated for 2 h at 37 °C. Absorbance at 450 nm was measured using CMax Plus (Molecular Devices, LLC). Each sample was tested in triplicate to obtain the average.

### 4.14. Wound Healing Assay

In brief, 6 × 10^5^ PC-9 or H1299 cells were seeded in 6-well plates for 24 h and then transfected with 80 pmol siControl, siLAD1 #1, or siLAD1 #2. About 48 h later (cells confluence reached 100%), 200 μL sterile pipette tips were used to scratch the wound uniformly. Then, the wells were washed with PBS to remove cell debris and replaced with fresh serum-free RPMI-1640. After 0/24/48/72 h, images at specific wound sites were taken under a microscope (Olympus CKX53) and the width of the wound was measured by Image J software. Each experiment was conducted in triplicate.

### 4.15. Colony Formation Assay

After 24 h following transfection with siControl, siLAD1 #1, or siLAD1 #2, PC-9 or H1299 cells were plated at 200 cells/well in 6-well plates for 2–3 weeks in RPMI-1640 supplemented with 10% FBS and 1% PS at 37 °C with 5% CO_2_. Then, the cells were washed with PBS and fixed in cold methanol for 30 min. Subsequently, 0.5% (*w/v*) crystal violet was used to stain the fixed colonies for 30 min at room temperature. Residual crystal violet was rinsed away with PBS. Then, the areas of cell colonies were analyzed by Image J software. Each experiment was repeated three times.

### 4.16. Animal Experiments

Six-week-old Balb/c nude mice were purchased from GemPharmatech and maintained at the SPF facility at CASHIPS. All animal studies were conducted according to protocols approved by the Ethical Committee of Experimental Animals of CASHIPS (DW-2020-13; data of approval, 8 April 2020). Mice were grafted with 5 × 10^5^ WT or LAD1-knockout PC-9 cells (sgLAD1 #1 and #2 mixed at 1:1) by subcutaneous injection into the right flank. Tumor volume was measured every three days with a vernier caliper and the volume was calculated according to the following: volume = π/6 × L × W2. After 15 days, 20 days later, all the mice were sacrificed and the tumors were excised.

### 4.17. Statistical Analysis

Differences in the expression of LAD1 in public datasets were compared by one-way ANOVA, and differences in clinical information and immune checkpoint inhibitor responses between the two different subgroups were compared by the chi-squared test. All data involved in the experiments were analyzed using GraphPad Prism 5 software or R software. Data are shown as means ± (standard deviations) SDs. Differences between samples were calculated by Student’s t-test. * *p* < 0.05, ** *p* < 0.01, *** *p* < 0.001, and *p* > 0.05 were considered not significant (ns). All experiments were performed at least 3 times.

## 5. Conclusions

In this paper, through bioinformatics combined with molecular biology, cell, and animal experiments, we proved that the frequent overexpression of LAD1 is a poor prognostic factor for LUAD, and the downregulation of LAD1 expression can inhibit the proliferation of lung adenocarcinoma cells in vitro and in vivo. We also innovatively proposed that LAD1 may assign a “K-Ras addiction” phenotype to LUAD tumor cells, and EGF signal activation closely related to lung cancer may belong to one of the carcinogenic signaling pathways. Therefore, the intervention of LAD1 could inhibit both EGF and K-Ras signals, thus inhibiting LUAD cell proliferation more effectively. Our work suggests that LAD1 is an important prognostic biomarker for LUAD and has the potential to be a therapeutic target for LUAD patients, deserving further attention and exploration.

## Data Availability

The datasets presented in this study can be found in online repositories, as mentioned in the section “Materials and Methods”. All data related to this study are available from the corresponding author upon reasonable request.

## Supplementary Materials

The following supporting information can be downloaded at: https://www.mdpi.com/article/10.3390/molecules28031103/s1, Figure S1: Detection of the IC50 of lung cancer cell lines to Gefitinib; Figure S2: Detection of the IC50 of lung cancer cell lines to K-Ras inhibitor 9.

## Abbreviations

AUC: area under the curve; CCTCC: China Center for Type Culture Collection; CPTAC: clinical proteomic tumor analysis consortium; EGA: European Genome-phenome Archive; EGFR: epidermal growth factor receptor; FBS: fetal bovine serum; GO: gene ontology, GSVA: Gene Set Variation Analysis; IHC: immunohistochemistry; KEGG: Kyoto Encyclopedia of Genes and Genomes; LAD1: Ladinin-1; LUAD: lung adenocarcinoma; LUSC: lung squamous cell carcinoma; LCLC: large-cell lung cancer; NSCLC: non-small-cell lung cancer; OS: overall survival; PFS: progression-free survival; PI: propidium iodide; PPS: post-progression survival; PS: penicillin–streptomycin; PTX: paclitaxel; RFS: relapse-free survival; ROC: receiver operating characteristic; SCLC: small-cell lung cancer; SEK: six serine–glutamate–lysine; ssGSEA: single-sample gene set enrichment analysis; TCGA: The Cancer Genome Atlas.
