# Supplementary material for: The Tumorigenic Effect of the High Expression of Ladinin-1 in Lung Adenocarcinoma and Its Potential as a Therapeutic Target
Source: Molecules. 2023 Jan 22;28(3):1103. doi: 10.3390/molecules28031103 (PMC9919345; doi:10.3390/molecules28031103)
Supplement: Supplementary file 1 [file molecules-28-01103-s001.zip › molecules-2047073-supplementary.pdf]

Supplementary

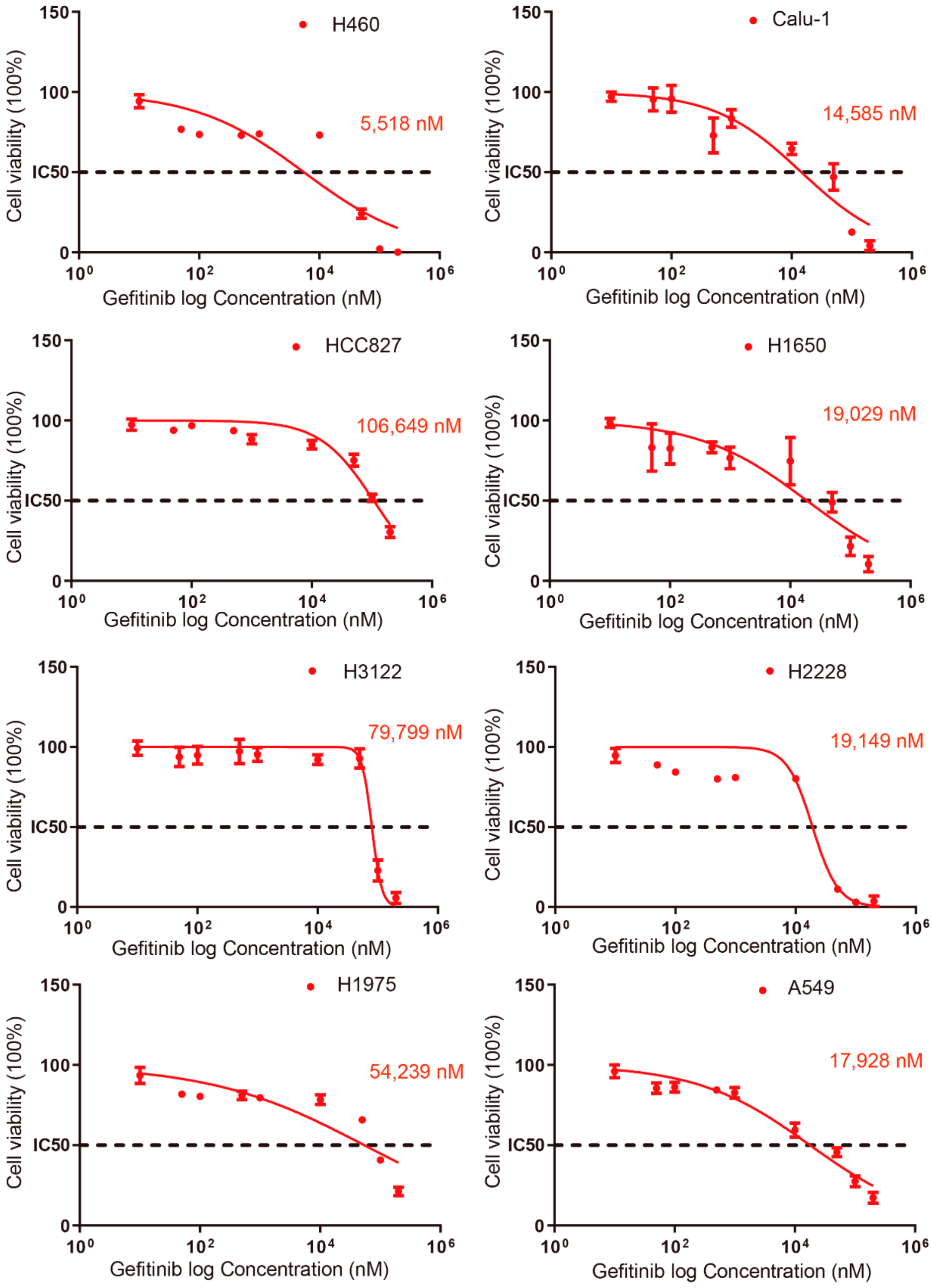

Figure S1. Detection IC50 of lung cancer cell lines to Gefitinib. Cells were seeded into 96-well plates at a number of 5000 per well for 24 h, then various concentrations of Gefitinib were added and incubated for another 24 h. Cell viability were detected by

CCK8 assay and the IC50 values were generated by GraphPad Prism 5. Each experiment was repeated three times. Error bars, means ± SD (*p < 0.05, **p < 0.01, ***p < 0.001 vs. control, n = 3 independent experiments).

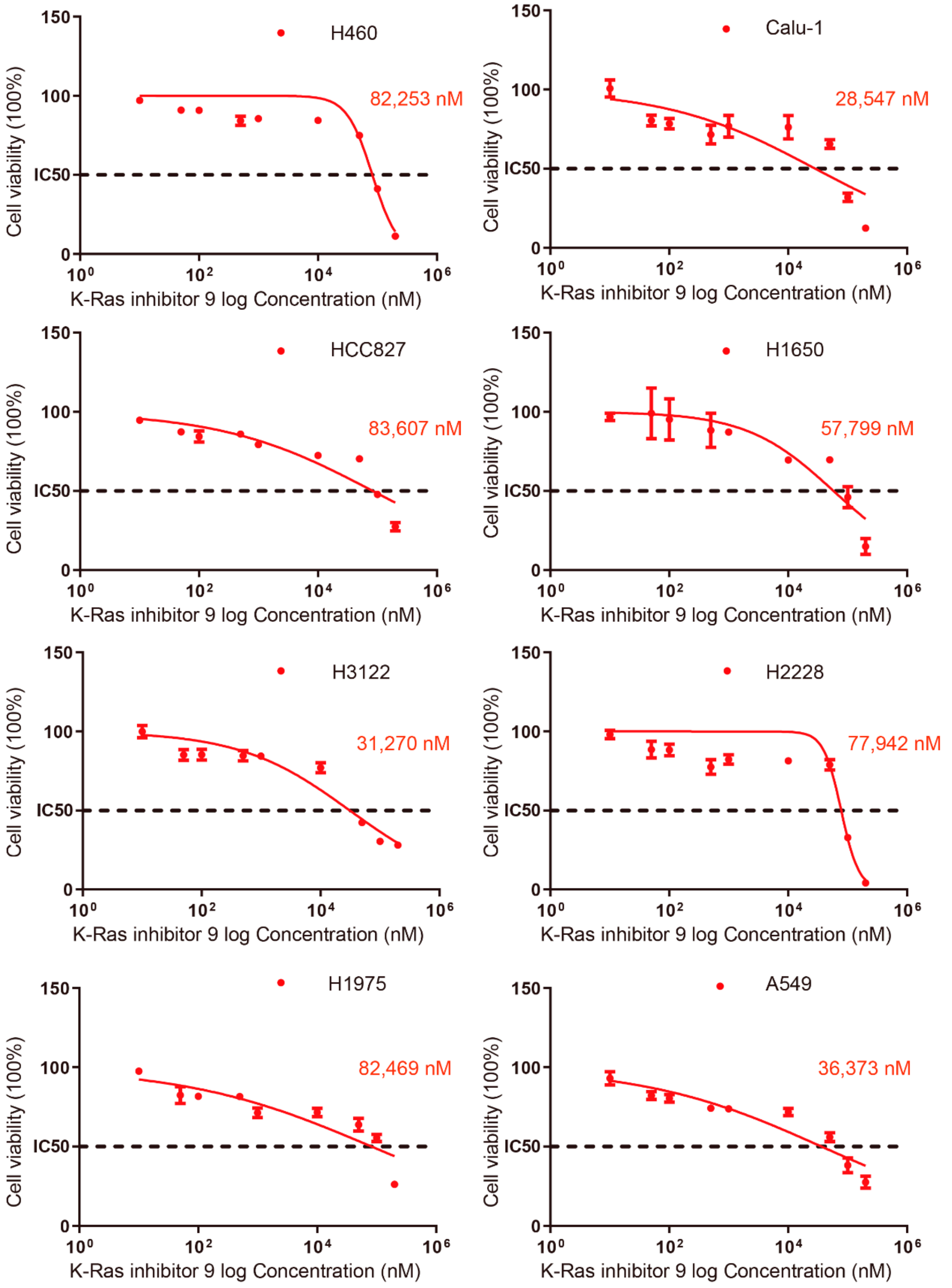

Figure S2. Detection IC50 of lung cancer cell lines to K-Ras inhibitor 9. Cells were

seeded into 96-well plates at a number of 5000 per well for 24 h, then various concentrations of K-Ras inhibitor were added and incubated for another 24 h. Cell viability were detected by CCK8 assay and the IC50 values were generated by GraphPad Prism 5. Each experiment was repeated three times. Error bars, means ± SD (*$p < 0.05$, **$p < 0.01$, ***$p < 0.001$ vs. control, n = 3 independent experiments).
